# *Egr1* loss-of-function promotes beige adipocyte differentiation and activation specifically in inguinal subcutaneous white adipose tissue

**DOI:** 10.1038/s41598-020-72698-w

**Published:** 2020-09-28

**Authors:** Marianne Bléher, Berbang Meshko, Isabelle Cacciapuoti, Rachel Gergondey, Yoann Kovacs, Delphine Duprez, Aurore L’Honoré, Emmanuelle Havis

**Affiliations:** 1 Sorbonne Université, 75005 Paris, France; 2 Institute of Biology Paris Seine-Developmental Biology Laboratory, Inserm U1156, French National Centre for Scientific Research (CNRS) UMR7622, 75005 Paris, France; 3 Institute of Biology Paris Seine-Integrative Cellular Ageing and Inflammation, French National Centre for Scientific Research (CNRS) UMR8256, 75005 Paris, France; 4 Inovarion, Paris, France

**Keywords:** Developmental biology, Stem cells

## Abstract

In mice, exercise, cold exposure and fasting lead to the differentiation of inducible-brown adipocytes, called beige adipocytes, within white adipose tissue and have beneficial effects on fat burning and metabolism, through heat production. This browning process is associated with an increased expression of the key thermogenic mitochondrial uncoupling protein 1, Ucp1. Egr1 transcription factor has been described as a regulator of white and beige differentiation programs, and Egr1 depletion is associated with a spontaneous increase of subcutaneous white adipose tissue browning, in absence of external stimulation. Here, we demonstrate that *Egr1* mutant mice exhibit a restrained *Ucp1* expression specifically increased in subcutaneous fat, resulting in a metabolic shift to a more brown-like, oxidative metabolism, which was not observed in other fat depots. In addition, *Egr1* is necessary and sufficient to promote white and alter beige adipocyte differentiation of mouse stem cells. These results suggest that modulation of *Egr1* expression could represent a promising therapeutic strategy to increase energy expenditure and to restrain obesity-associated metabolic disorders.

## Introduction

White adipose tissue (WAT) plays a crucial role in energetic homeostasis regulation through the storage of excess energy in the large central vacuole of white adipocytes^1^. WAT also performs metabolic functions and secretes several hormones such as Resistin (*Retn*)^1–3^ and Leptin (*Lep*) which regulates body weight, lipid and glucose metabolism^4–6^. WAT is located in diverse sites throughout the body, such as the subcutaneous inguinal (SC-WAT), perigonadal (GAT), mesenteric (MAT) and perirenal (PAT) depots^7,8^. While these depots are collectively named white adipose tissue, they have different developmental origins: SC-WAT, GAT and MAT derive from lateral plate mesoderm in female mice whereas PAT derive from the somitic mesoderm^8^. These different origins could contribute to differences in the physiology of fat depots.

In contrast, brown adipose tissue (BAT) has a critical thermogenic function thanks to its property to dissipate excess energy through heat production^9^. BAT depots are derived from the somitic mesoderm^8^ and are located in the inter-scapular region in mice and in both neck and supraclavicular regions in humans^9^. Brown adipocytes are smaller than white adipocytes. They exhibit multiple small lipid vacuoles and large number of mitochondria involved in heat production through the constitutive activation of the thermogenic uncoupling protein 1 (*Ucp1*)^10–12^.

A third type of adipocyte, called beige adipocyte, has been described within WAT^9,13^. Morphological and transcriptomic analyses show that brown and beige adipocytes are remarkably similar and express the same thermogenic markers, including *Ucp1*^14^. In mice, beige adipocytes differentiation occurs in response to long term cold exposition, fasting or exercise through a process referred to as browning^9,15^. White, beige and brown adipocytes have been shown to differentiate in vitro from adipose-derived mesenchymal stem/stromal cells (ASC) isolated from lipoaspirates^16–19^. In this context, we previously demonstrated that mice lacking *Egr1* expression exhibit spontaneous browning of the inguinal subcutaneous white adipose tissue without external stimulation^20^. Egr1 is a multi-faceted transcription factor involved in the development and homeostasis of several organs^20–23^. Increased *Egr1* expression has been linked to obesity in both humans and murine models^22,23^. In contrast, *Egr1* knock-out mice display an increased energy expenditure and are protected from high fat diet-induced obesity and obesity-associated pathologies^23,24^. The browning of *Egr1*^−/−^ mice is associated with up-regulation of beige adipocyte gene expression such as *Ucp1*, *Cebpb*, *Cidea* and *Cox8b*, together with a decreased expression of the white adipocyte marker *Lep*. Egr1 transcription factor can alternatively acts as a repressor or an activator depending of its interacting partners^21^. In this context, we previously showed that in SC-WAT, both *Ucp1* and *Cebpb* expressions are repressed by Egr1 through direct binding to their promoters^20^. Loss of *Egr1* can therefore spontaneously induce SC-WAT browning through the release of this repression and the consequent activation of beige differentiation by *Ucp1* and *Cebpb*^20^. In this study, we get further to first analyze the consequences of *Egr1* knock-out on *Ucp1* expression in all adipose tissue depots. Then, we tested whether *Ucp1* up-regulation in WAT comes with a shift to a BAT-like oxidative metabolism. Interestingly, we observed that *Ucp1* up-regulation in mutant *Egr1*^−/−^ adipose tissue was restricted to subcutaneous inguinal SC-WAT and associated with an increased oxygen consumption rate in this fat pad. To further characterize the role of *Egr1* in SC-WAT browning, adipose stem cells (mASC) were isolated from control *Egr1*^+*/*+^ and mutant *Egr1*^*−/−*^ SC-WAT and compared for their ability to differentiate into white or beige adipocytes in vitro. We observed that *Egr1* was necessary to promote white adipocyte differentiation and repress beige differentiation of mASC. *Egr1* gain-of-function experiments performed in C3H10T1/2 mesenchymal stem cells confirmed the positive role of *Egr1* on white adipocyte differentiation and its negative role on beige differentiation. Collectively, our results indicate that *Egr1* favors white adipocyte differentiation and represses SC-WAT browning. *Egr1* may thus become a promising target to combat obesity.

## Results and discussion

### *Egr1*^*−/−*^ mice display spontaneous increase of *Ucp1* expression specifically in SC-WAT

**Figure 1:**
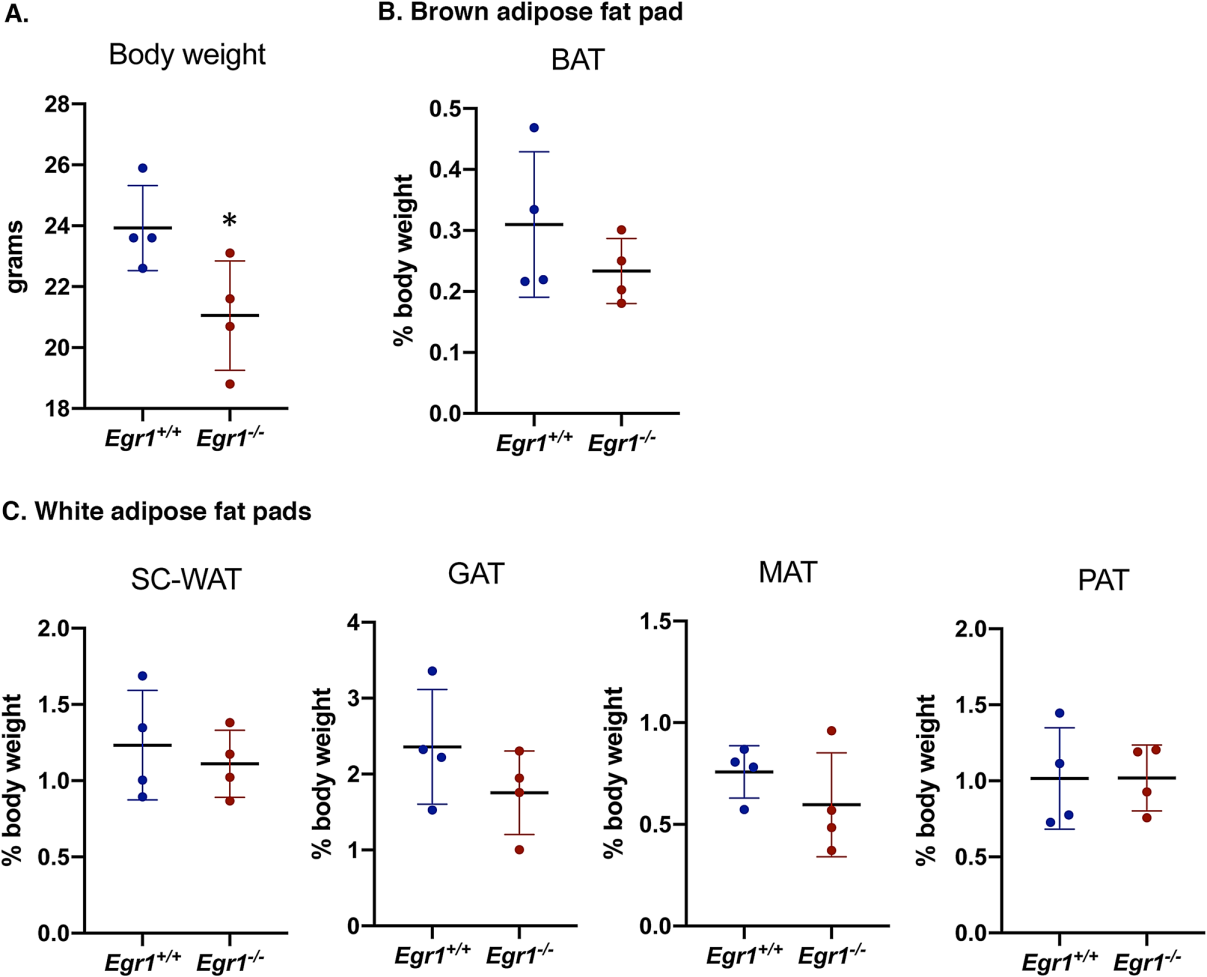
*Egr1* loss-of-function does not affect fat pad percentage of body weight. (**A**) Whole body, (**B**) Brown adipose tissue (BAT), (**C**) Inguinal sub-cutaneous adipose tissue (SC-WAT), gonadal adipose tissue (GAT), mesenteric adipose tissue (MAT) and perirenal adipose tissue (PAT) of 8-month-old *Egr1*^+*/*+^ and *Egr1*^*−/−*^ female mice were weighted. Fat pad weights were represented as a percentage of body weight. Experiments were performed with n = 4 for each genotype. Error bars represent the mean + /− standard deviations. The *p* values were obtained using the Student t test, with *p < 0.05.

**Figure 2:**
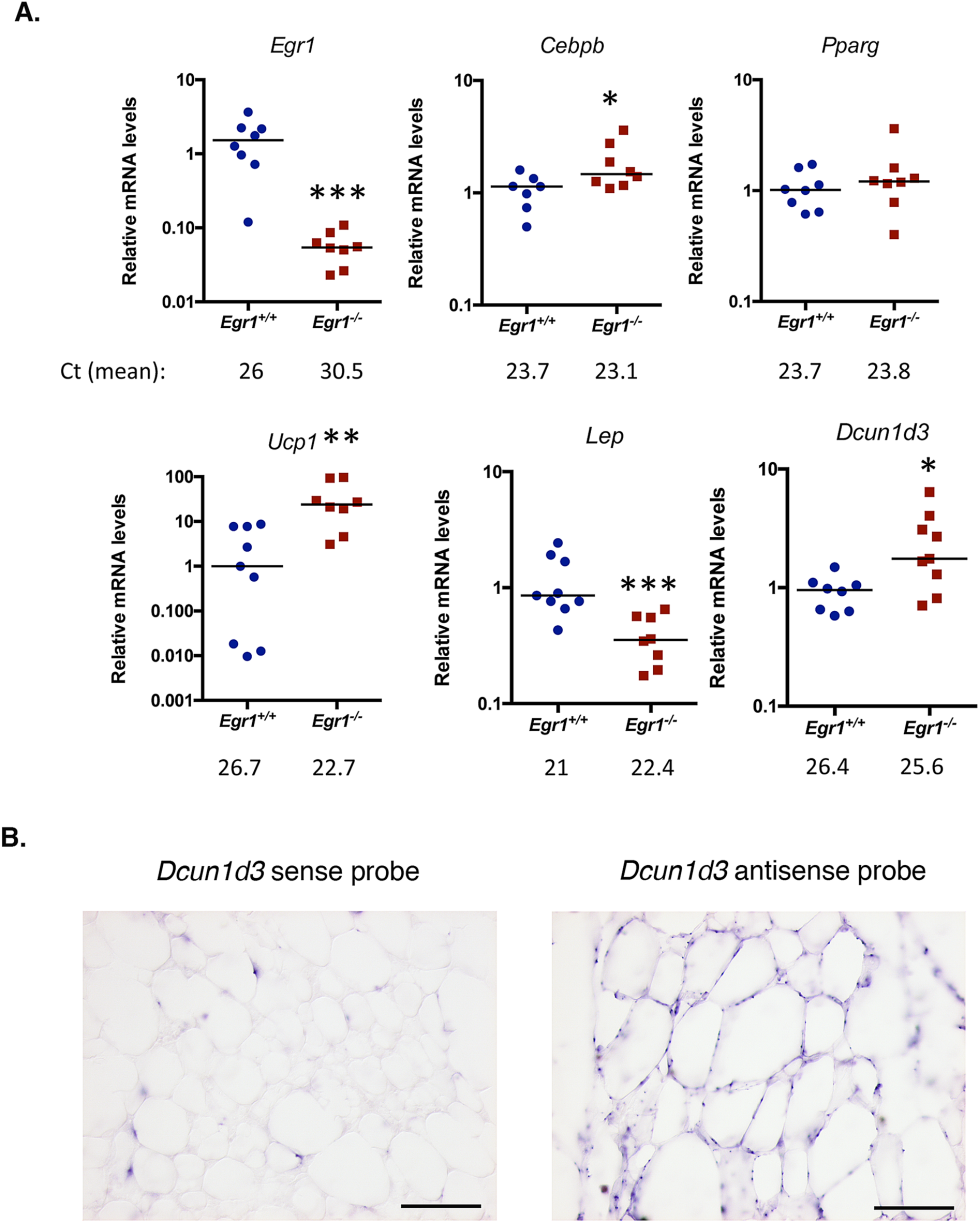
*Egr1* loss-of-function increases *Ucp1* expression in SC-WAT. (**A**) SC-WAT of 8-month-old *Egr1*^+*/*+^ and *Egr1*^*-/−*^ mice were used for RNA purification. RNA samples were analysed by RT-qPCR for *Egr1*, *Cebpb*, *Pparg*, *Ucp1*, *Lep* and *Dcun1d3* expression. Experiments were performed with n = 8 animals for each genotype. Transcripts are shown relative to the level of *Rp18S* transcripts, with control *Egr1*^+*/*+^ being normalized to 1. The relative mRNA levels were calculated using the 2^−∆∆Ct^ method. The *p* values were obtained using the Mann-Withney test, with *p < 0.05, **p  < 0.01, ***p < 0.001. (**B**) SC-WAT from wild-type mice was longitudinally cryosectioned. 8 µm sections were hybridized with the DIG-labeled sense and antisense probes for *Dcun1d3*. Scale bars: 100 µm.

*Egr1* loss-of-function in postnatal and 4-month-old mice leads to a reduced body weight despite similar weight of SC-WAT fat pads in control *Egr1*^+*/*+^ and mutant *Egr1*^*−/−*^ mice^20^. Significant body weight reduction was confirmed in 8-month-old *Egr1*^*-/-*^ mice (Fig. 1A). In addition, all isolated white subcutaneous (SC-WAT), gonadic (GAT), mesenteric (MAT) and perirenal (PAT) and brown (BAT) fat pads display similar percentage of body weight in control *Egr1*^+*/*+^ and mutant *Egr1*^*-/-*^ mice (Fig. 1B, C). These observations indicate that lower body weight observed in mutant *Egr1*^*-/-*^ mice is not due to a reduced adipose tissue mass, and suggest that *Egr1* loss-of-function reduces body weight through unknown global metabolic processes. *Egr1* loss-of-function has been shown to induce spontaneous browning of SC-WAT, associated with increased *Ucp1* expression, without external stimulation^20^. Here, we quantified the expression of *Egr1*, *Cebpb*, *Pparg*, *Ucp1*, *Lep* and *Dcun1d3* in five different adipose tissue depots from control *Egr1*^+*/*+^ and *Egr1*^*−/−*^ mice. As expected, *Egr1* was nearly undetectable in mutant *Egr1*^*-/-*^ fat pads (Fig. 2A, Supplementary Figs. 1 and 2A–C). A significant increase of *Cebpb* and *Ucp1* expression was observed in mutant *Egr1*^*−/−*^ SC-WAT compared to control, associated with a decrease of *Lep* expression (Fig. 2A). In contrast, *Cebpb*, *Ucp1* and *Lep* expression were not altered in the other 4 fat pads in mutant versus control (Supplementary Fig. 1 and 2A–C) except in MAT in which *Lep* expression was lower in mutants. These observations are consistent with previous results showing the absence of spontaneous browning in BAT and GAT from *Egr1*-deleted mice^23^. Our results indicate that *Egr1* deficiency induces a browning phenotype restricted to SC-WAT, and suggest that *Egr1* is required to repress SC-WAT browning in standard housing conditions. SC-WAT browning in mutant *Egr1*^*−/−*^ mice is characterized by an increased number of beige adipocytes at the expense of white adipocytes^20^. Taken together, our results suggest that Egr1 represses *Cebpb*, *Ucp1* and activates *Lep* expression in SC-WAT, which is in accordance with previous results showing recruitment of Egr1 to their promoters^20,25^. Decreased *Lep* expression in MAT depot in *Egr1*-null mice (MAT, Supplementary Fig. 2B) suggests that Egr1 can activate *Lep* independently of the browning phenotype. At last, as previously observed, *Pparg* expression was not affected by *Egr1* loss-of-function in SC-WAT^20^ neither in the 4 other fat pads (Fig. 2A, Supplementary Figs. 1 and 2A–C).

*Dcun1d3* has been shown upregulated in mutant *Egr1*^*-/-*^ SC-WAT^20^. *Dcun1d3* is expressed in adipocytes within adipose tissue (Fig. 2B), however its role has not been elucidated yet. While *Dcun1d3* was present in all adipose depots in controls (Fig. 2A, Supplementary Fig. 1 and 2A–C), its expression was specifically upregulated in *Egr1*-deficient SC-WAT (Fig. 2A, Fig. 2A, Supplementary Figs. 1 and 2A–C). This suggests that *Dcun1d3* may be related to SC-WAT browning in mutant *Egr1*^*−/−*^ mice.

Taken together, these results indicate that *Egr1* loss-of-function increases *Cebpb*, *Ucp1* and *Dcun1d3* expression and decreases *Lep* expression in SC-WAT, resulting in spontaneous browning in this specific adipose tissue depot. One hypothesis could be that Egr1 interacts with specific co-regulators in fat pads, leading to the regulation of different genetic programs in each depot. The co-factors allowing Egr1 to regulate the browning program might be specifically expressed in SC-WAT.

### *Egr1* loss-of-function induces a metabolic shift in white adipose tissue, leading to a BAT-like oxidative metabolism

**Figure 3:**
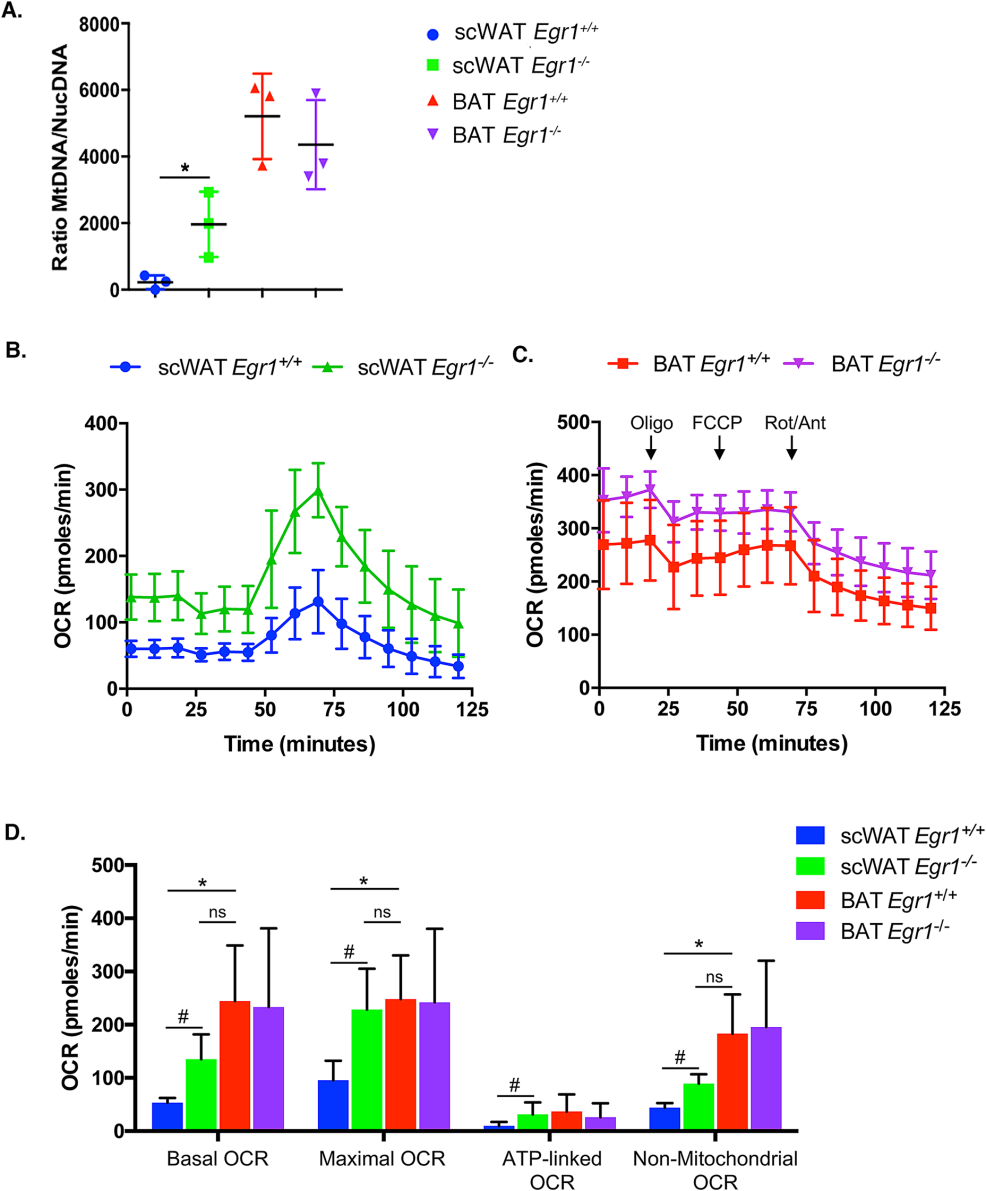
*Egr1* loss-of-function induces a metabolic shift in white adipose tissue, leading to a BAT-like oxidative metabolism. (**A**) WAT and BAT of 8-month-old control *Egr1*^+*/*+^ and mutant *Egr1*^*−/−*^ female mice were dissected and used for DNA purification. Mitochondrial (*Cyt B*) and nuclear (*Ndufv1*) genes were quantified by qPCR and histogram represents their ratio. Error bars represent the means + standard deviations with n = 3 animals for each genotype, *p < 0.05. (**B, C**) Mitochondrial respiration measured by oxygen consumption rate (OCR) in basal conditions and after sequential addition of Oligomycin, FCCP, and a mix of Rotenone/Antimycin were simultaneously recorded on SC-WAT (B) and BAT (C) tissues, freshly dissected from 8-month-old control *Egr1*^+*/*+^ and mutant *Egr1*^*−/−*^ female mice. (**D**) Histogram represents the basal OCR (determined as the difference between OCR before oligomycin and OCR after rotenone/antimycin A), maximal OCR (difference between OCR after FCCP and OCR after rotenone/antimycin A), ATP-linked OCR (difference between OCR before and after oligomycin), and the non-mitochondrial OCR (OCR after rotenone and antimycin A treatment) calculated from data obtained in A. Error bars represent the means + standard deviations with n = 3 animals for each genotype, *p < 0.05.

We then investigated whether the brown transcriptomic signature observed in mutant *Egr1*^*−/−*^ SC-WAT was correlated with metabolic modifications. While control *Egr1*^+*/*+^ SC-WAT is characterized by a low mitochondrial mass, marked by a low mitochondrial/nuclear DNA ratio, we observed an increased mitochondrial mass in mutant SC-WAT (Fig. 3A), with a ratio being much similar to that of BAT. Such increase is specific to this tissue and was not observed in any other tested fat tissues (Supplementary Fig. 3). To examine whether this increased mitochondrial mass has functional metabolic consequences, we assessed oxygen consumption rate (OCR) of BAT, SC-WAT and GAT freshly dissected from control and mutant mice. Using Seahorse XFe24 analyzer, we showed that while *Egr1* loss-of-function does not affect GAT (Supplementary Fig. 3B, C) or BAT OCR (Fig. 3B, C, D), it leads to an increase of all markers of oxidative capacity, including basal and maximal OCR, ATP-linked OCR and non-mitochondrial OCR. Importantly, all rates quantified in mutant *Egr1*^*−/−*^ SC-WAT were close to that obtained with BAT, demonstrating a metabolic shift of *Egr1*^*−/−*^ SC-WAT towards a BAT-like oxidative metabolism. As expected, this increase of oxidative capacity does not lead to altered ROS production, as indicated by similar carbonylation protein levels (Supplementary Fig. 3D), thus demonstrating that the browning process observed in *Egr1* mutant occurs in physiological conditions.

Our previous study has revealed a spontaneous browning of the SC-WAT in *Egr1* mutant, with *Ucp1* expression being up-regulated in absence of external stimulation^20^. We now show that this browning also exerts at physiological level with consequences on metabolic and bio-energetic activity of SC-WAT. Our results reveal a shift of *Egr1*^*-/-*^ SC-WAT towards a BAT-like oxidative metabolism and are correlated with previous studies establishing a link between increased *Egr1* expression and obesity^22,23^. They suggest a central role of *Egr1* in regulation of SC-WAT homeostasis in the adult.

### *Egr1* is necessary to promote white and to repress beige adipocyte differentiation of mouse adipose stem cells

**Figure 4:**
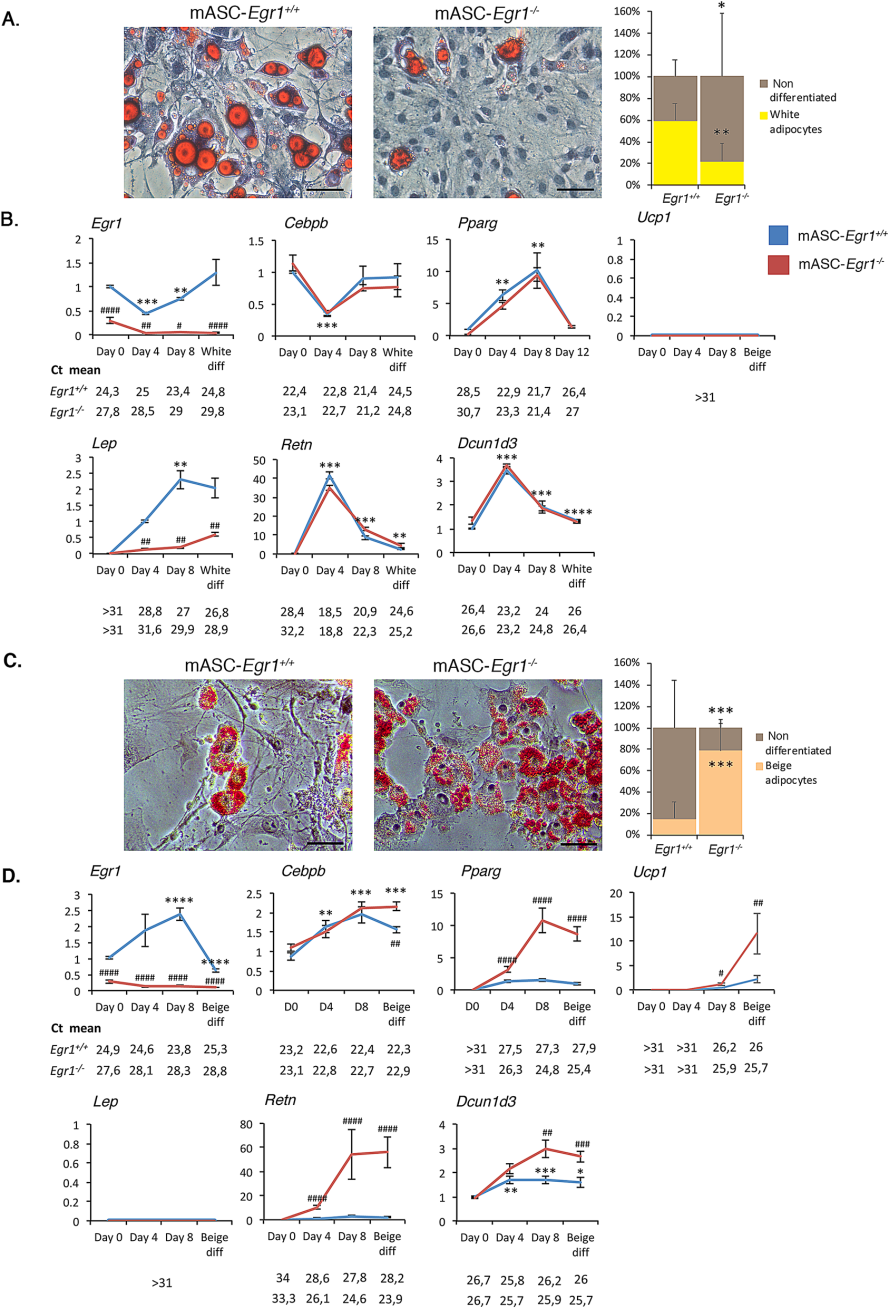
*Egr1* is necessary to promote white adipocyte differentiation and prevent beige differentiation of mouse adipose stem/stromal cells. (**A**–**F**) Control mASC-*Egr1*^+*/*+^ and mutant mASC-*Egr1*^*-/-*^ were subjected to white (**A**,**C**,**D**) or beige (**B**,**E**,**F**) differentiation for 12 or 10 days, respectively. (**A, C**) Non-differentiated and white (**A**, right panel) or beige (**C**) adipocytes were stained with Oil Red O and Hematoxilin after white (**A**, left panel) or beige (**C**, left panel) differentiation and counted (**A** and **C**, right panels). For each genotype, results are expressed as a percentage of cells differentiated or not, counted from 7 pictures at Days 0 and 8 after full differentiation. Scale bars, 50 µm. Error bars show means + standard deviations, with *P < 0.05, **P < 0.01. The *p* values were obtained using the Mann–Whitney test. (**B, D**) After 0, 4, 8 and 12 days of culture in white (**B**) or beige (**D**) differentiation medium, cells were used for RNA purification. RNA samples were analysed by RT-qPCR analysis for *Egr1*, *Dcun1d3*, *Ucp1*, *Lep* and *Retn* expression in control mASC-*Egr1*^+*/*+^ and mutant mASC-*Egr1*^*−/−*^ cells. Transcripts are shown relative to the level of *Rplp0* and *18S* transcripts. The relative mRNA levels were calculated using the 2^−∆∆Ct^ method, with control being normalized to 1. For cells subjected to white differentiation, experiments were performed with n ≥ 5 independent cultures for each genotype (n = 12 at Day0, n = 6 at Day4, n = 5 at Day8 and n = 10 at Day12 for control mASC-*Egr1*^+*/*+^ cells; n = 11 at Day0, n = 6 at Day4, n = 6 at Day8 and n = 12 at Day12 for mutant mASC-*Egr1*^*−/−*^ cells). For cells subjected to beige differentiation, experiments were performed with n = 16 independent cultures for each timepoint and genotype. Error bars represent the mean + standard deviations. The *p* values were obtained using the Mann–Withney test. Asterisks * indicate the *p* values of gene expression levels in mASC-*Egr1*^+*/*+^ and mASC-*Egr1*^*−/−*^ compared to the first day of gene detection *P < 0.05, **P < 0.01, ***P < 0.001, ****P < 0.0001; # indicate the *p* values of gene expression levels in mASC-*Egr1*^+*/*+^ versus mASC-*Egr1*^*−/−*^, for each time point, ^#^*P* < 0.05, ^##^*P* < 0.01, ^###^*P* < 0.001, ^####^*P* < 0.0001.

To further analyze the role of *Egr1* in spontaneous browning of SC-WAT in mutants, we tested whether *Egr1* loss-of-function can alter the differentiation of SC-WAT-derived mouse adipose stem cells (mASC) into white and beige adipocytes. mASC were isolated from inguinal SC-WAT of control *Egr1*^+*/*+^ and mutant *Egr1*^*−/−*^ mice and subjected to white (Fig. 4A, B) or beige (Fig. 4C, D) adipogenic differentiation.

At the end of white adipogenic stimulation, 60% of control *Egr1*^+*/*+^ cells have acquired a white adipocyte phenotype, marked by the presence of large lipid droplets in the cytoplasm (Fig. 4A). In contrast, only 20% of mutant mASC-*Egr1*^*−/−*^ cells exhibited lipid droplets (Fig. 4A) suggesting that *Egr1* is necessary for white adipocyte differentiation. To confirm this hypothesis, we quantified the expression of adipocyte markers in control and mutant cells at different days after differentiation. As expected, *Egr1* is highly expressed during the entire white adipocyte differentiation of control mASC-*Egr1*^+*/*+^ cells, while not detected in mutant mASC-*Egr1*^*−/−*^ cells (Fig. 4B). *Cebpb* and *Pparg* encode transcription factors belonging to the core genetic program that trigger adipogenesis^26^. In both control mASC-*Egr1*^+*/*+^ and mutant mASC-*Egr1*^*−/−*^, *Cebpb* expression first decreases after 4 days of white differentiation and returns to basal level at the end of the process. *Pparg* expression continuously increases until day 8, and returns to basal level at the end of white differentiation. These results indicate that *Egr1* is not necessary to activate *Cebpb* and *Pparg* expression during white adipocyte differentiation. Since Egr1 has been described as a direct regulator of *Cebpb* expression by chromatin immunoprecipitation assays^20^, our observations suggest that absence of Egr1 could be compensated in mASC-*Egr1*^*-/-*^ cells to activate *Cebpb* expression, or that Egr1 is recruited to *Cebpb* promoter only as a transcriptional repressor of beige adipocyte differentiation^20^. The beige adipocyte marker *Ucp1* was not expressed either in control or mutant cells. During white adipocyte differentiation, *Lep*, a late marker of white adipocyte differentiation^27,28^, was significantly down-regulated in mutant mASC-*Egr1*^*-/-*^ cells compared to controls, which is consistent with the reduced white adipocyte differentiation observed in *Egr1*^*−/−*^ cells (Fig. 4A). Resistin (*Retn*) is an adipocyte-specific hormone secreted by WAT^3^. In control and mutant cells, *Retn* expression was absent at day 0, increased during white differentiation reaching a peak at day 4, and subsequently decreased during late differentiation (Fig. 4B). A similar *Retn* expression profile has been previously described during white adipocyte differentiation of 3T3-L1 preadipocytes^29^. This result indicates that *Egr1* may not regulate *Retn* expression or that the absence of *Egr1* was compensated. *Dcun1d3* displays a similar expression profile to *Retn* during white differentiation of control mASC-*Egr1*^+*/*+^ cells. In contrast to what we previously observed in vivo with the *Egr1*^*−/−*^ mutant (Fig. 2A), *Egr1* loss-of-function did not affect *Dcun1d3* expression during mASC white adipocyte differentiation (Fig. 4B). This result can be explained by the difference between in vivo analysis of a mixed tissue, and study of an in vitro homogenous cell population strictly directed towards white differentiation.

These observations demonstrate that while mutant mASC-*Egr1*^*−/−*^ cells express some markers at a similar level than in control cells (*Cebpb*, *Pparg*, *Retn* and *Dcun1d3,* Fig. 4B*)*, they present significant morphological (Fig. 4A) and transcriptomic (Fig. 4B, *Lep*) alterations in late markers of normal white adipocyte differentiation. This suggests that *Egr1* has a positive role on white adipocyte differentiation.

Following beige adipogenic stimulation, control mASC-*Egr1*^+*/*+^ cells displayed a low level (17%) of differentiation into beige adipocytes marked by the appearance of numerous small lipid droplets in their cytoplasm (Fig. 4C). In contrast, *Egr1* deletion led to a robust differentiation of 78% of mutant mASC-*Egr1*^*−/−*^ cells into beige adipocytes (Fig. 4C). This result shows that beige adipocyte differentiation in wild type mASC is a less efficient process compared to white differentiation (Fig. 4A, C). More importantly, this observation reveals that *Egr1* acts as a critical repressor of beige adipocyte differentiation. In wild type mASC, *Egr1* expression increases during the first time-points of beige adipocyte differentiation and decreases at the end of the process (Fig. 4C). In contrast to white adipocyte differentiation, *Cebpb* expression increases during wild type mASC beige adipocyte differentiation whereas *Pparg* expression remains constant from day 4 (Fig. 4D). Interestingly, *Egr1* loss-of-function significantly increases *Cebpb* and *Pparg* expression, suggesting that Egr1 is necessary to repress *Cebpb* and *Pparg* expression during beige adipocyte differentiation. As expected, *Ucp1* expression is significantly upregulated in mutant mACS-*Egr1*^*−/−*^ compared to control cells (Fig. 4D)*,* consistently with the browning phenotype of *Egr1*^*−/−*^ mice (Figs. 2 and 3)^20^. *Lep* is not expressed during beige adipocyte differentiation of both control and mutant mASC (Fig. 4D). Expression of *Retn* is detected during beige adipocyte differentiation (Fig. 4D), while at lower levels compared to white differentiation, which further suggests that *Retn* may be a generic marker of both white and beige adipocyte differentiation^13^. Similarly, mutant mASC-Egr1^−/−^ cells which exhibit a robust differentiation rate, visualized by accumulation of lipid droplets in the cytoplasm (78%, Fig. 4C), are characterized by higher *Retn* expression levels compared to wild type cells (Fig. 4D). This further confirms that *Retn* expression can be associated with beige adipocyte differentiation. *Dcun1d3* expression is increased during beige adipocyte differentiation (Fig. 4D). In addition, *Egr1* loss-of-function leads to a significant upregulation in *Dcun1d3* expression (Fig. 4D), suggesting that Egr1 represses *Dcun1d3* expression. This is similar to our in vivo results (Fig. 2B).

Altogether, our results indicate that *Egr1* is necessary to promote white adipocyte differentiation and to repress beige adipocyte differentiation of mASC. Consistent with the cell differentiation phenotypes, Egr1 is necessary to activate *Lep* during white differentiation and to repress *Ucp1* during beige adipocyte differentiation. *Retn* and *Dcun1d3* are expressed during both white and beige adipocyte differentiation of mASC, suggesting a generic role of these factors during adipocyte differentiation (Fig. 7). Interestingly, *Egr1* loss-of-function specifically affects the expression of the generic adipocyte differentiation markers *Cebpb*, *Pparg*, *Retn* and *Dcun1d3* during beige adipocyte differentiation, which confirms the negative role of *Egr1* on beige differentiation suggested by in vivo data (Figs. 2 and 3). The positive role of *Egr1* on in vitro white adipocyte differentiation is consistent with our in vivo results (Figs. 2 and 3) and with studies correlating *Egr1* overexpression with obesity^22,23^.

### *Egr1* is sufficient to promote white and to repress beige adipocyte differentiation of C3H10T1/2 mesenchymal stem cells

**Figure 5:**
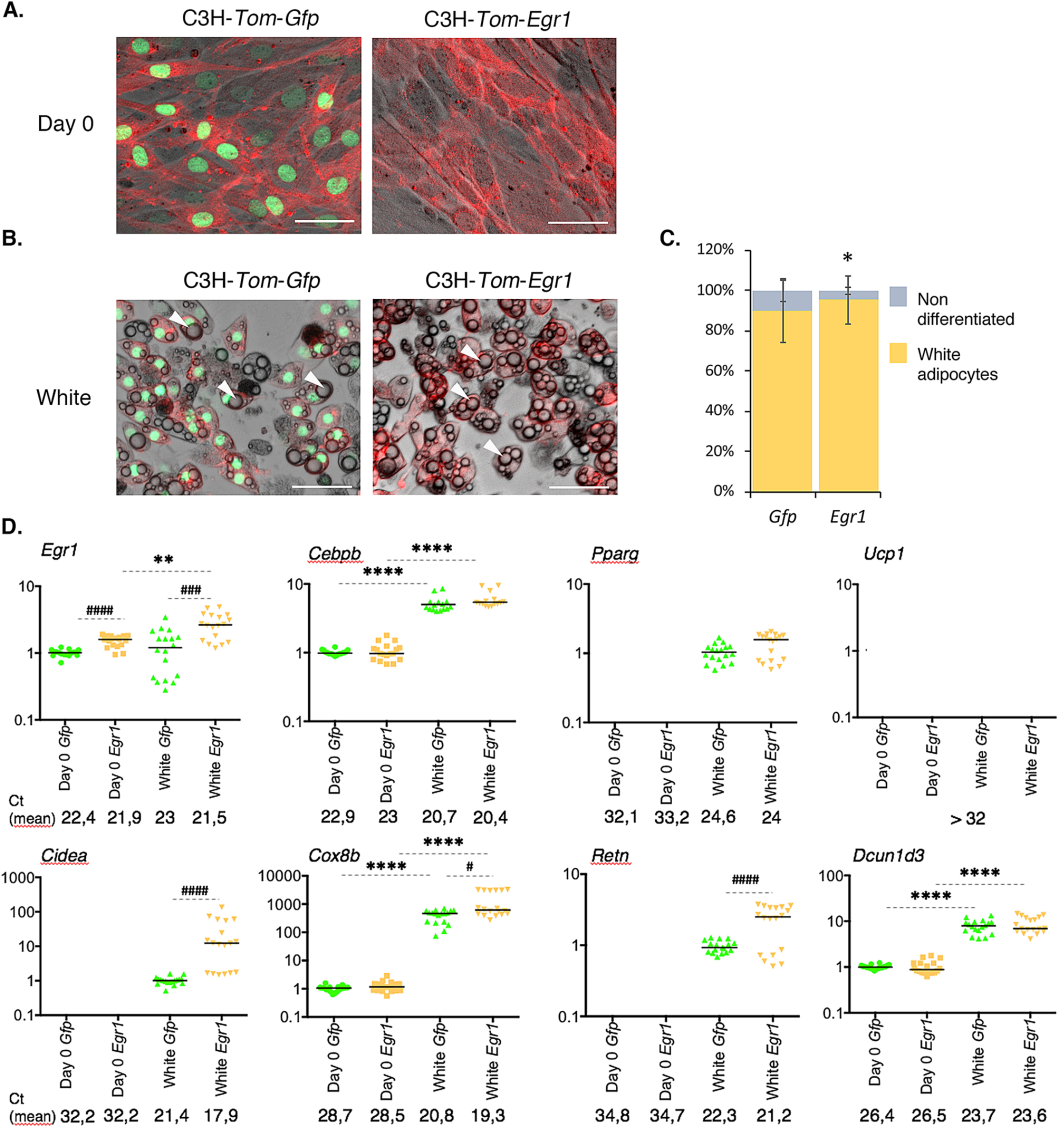
*Egr1* is sufficient to promote white adipocyte differentiation in a multipotent cell line. (**A**–**D**) Control C3H-*Tom*-*Gfp* and *Egr1*-overexpressing C3H-*Tom*-*Egr1* cells were subjected to white differentiation. At day 0 (**A**) and at the end of the differentiation process (**B**), apotome images were obtained using brightfield, green (Gfp) and red (Tom) channels, merged, and a representative image is shown for each condition. White arrowheads indicate lipid droplets. Scale bars, 50 µm. (**C**) C3H-*Tom*-*Gfp* and C3H-*Tom*-*Egr1* cells submitted to white adipogenic medium were counted from n = 7 pictures and expressed as a percentage of total cells differentiated or not for each condition or as total number of cells per arbitrary unit area. Error bars represent the means +/- standard deviations, and *p* values were obtained using the Mann–Whitney test comparing differentiated and non-differentiated C3H-*Tom*-*Gfp* and C3H-*Tom-Egr1* cells, *P < 0.05. (**D**) At Day 0 or at the end of white differentiation, cells were used for RNA purification. RNA samples were analysed by RT-qPCR analysis for *Egr1*, *Cebpb*, *Pparg*, *Ucp1*, *Cidea*, *Cox8b*, *Retn* and *Dcun1d3* expression in control C3H-*Tom*-*Gfp* and *Egr1*-overexpressing C3H-*Tom*-*Egr1* cells. Transcripts are shown relative to the level of *18S* transcripts. The relative mRNA levels were calculated using the 2^−∆∆Ct^ method, with control being normalized to 1. Experiments were performed with n = 18 independent cultures for C3H-*Tom*-*Gfp* and C3H-*Tom*-*Egr1* cells. * indicate the *p* values of gene expression levels in C3H-*Tom*-*Gfp* or C3H-*Tom*-*Egr1* at the first day of gene detection versus differentiation, with **P < 0.01, ****P < 0.0001; # indicate the *p* values of gene expression levels in C3H-*Tom*-*Gfp* versus C3H-*Tom*-*Egr1* for each time point, with ^#^*P* < 0.05, ^###^*P* < 0.001, ^####^*P* < 0.0001.

**Figure 6:**
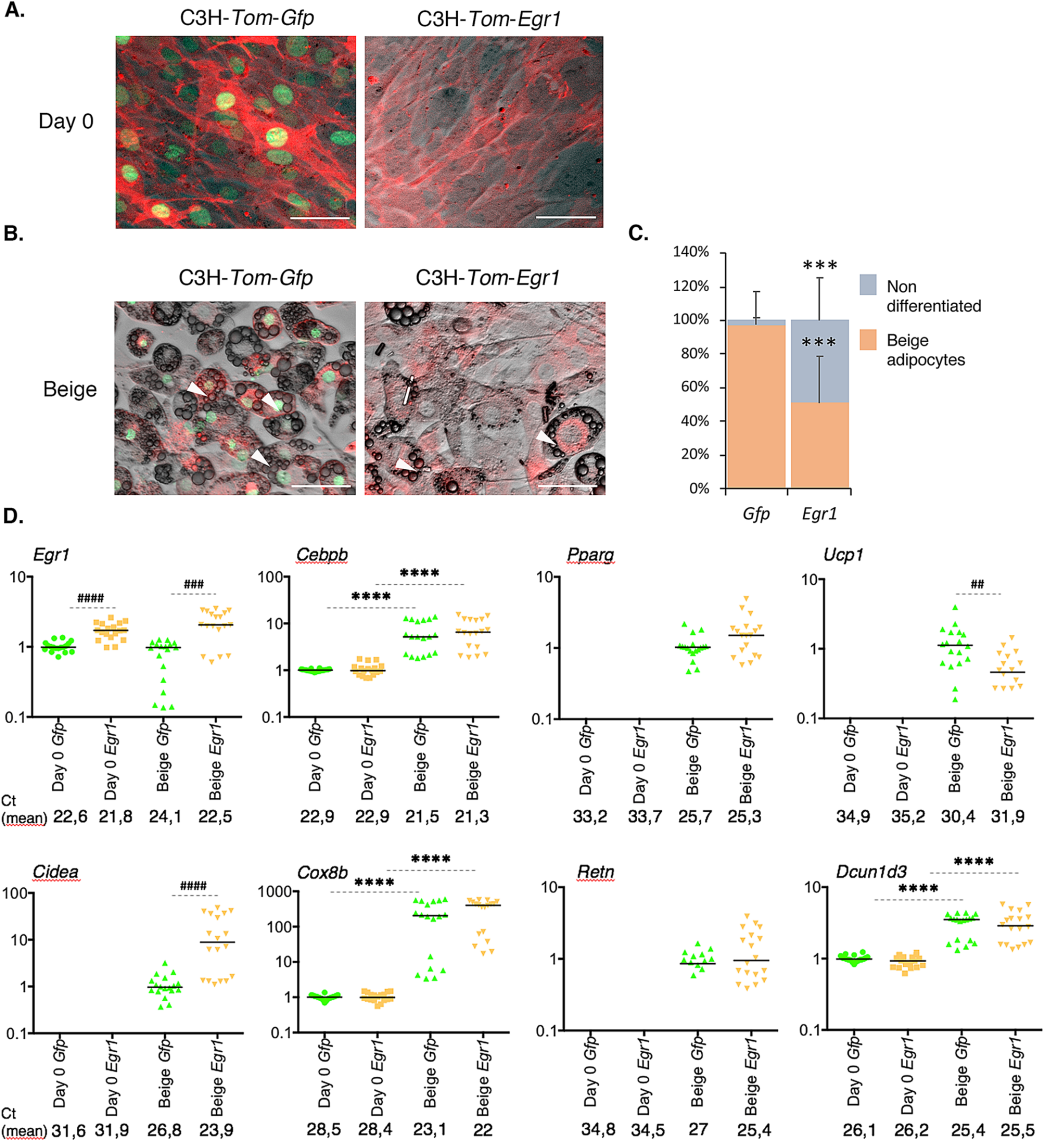
*Egr1* is sufficient to reduce beige adipocyte differentiation in a multipotent cell line. (**A**–**D**) Control C3H-*Tom*-*Gfp* and *Egr1*-overexpressing C3H-*Tom*-*Egr1* cells were subjected to beige differentiation. At day 0 (**A**) and at the end of the differentiation process (**B**), apotome images were obtained using brightfield, green (Gfp) and red (Tom) channels, merged, and a representative image is shown for each condition. White arrowheads indicate lipid droplets. Scale bars, 50 µm. (**C**) C3H-*Tom*-*Gfp* and C3H-*Tom*-*Egr1* cells submitted to beige adipogenic medium were counted from n = 6 pictures and expressed as a percentage of total cells differentiated or not for each condition or as total number of cells per arbitrary unit area. Error bars represent the means + standard deviations, and *p* values were obtained using the Mann–Whitney test comparing differentiated and non-differentiated C3H-*Tom*-*Gfp* and C3H-*Tom-Egr1* cells, ***P < 0.001. (**D**) At Day 0 or at the end of beige differentiation, cells were used for RNA purification. RNA samples were analysed by RT-qPCR analysis for *Egr1*, *Cebpb*, *Pparg*, *Ucp1*, *Cidea*, *Cox8b*, *Retn* and *Dcun1d3* expression in control C3H-*Tom*-*Gfp* and *Egr1*-overexpressing C3H-*Tom*-*Egr1* cells. Transcripts are shown relative to the level of *18S* transcripts. The relative mRNA levels were calculated using the 2^−∆∆Ct^ method, with control being normalized to 1. Experiments were performed with n = 18 independent cultures for C3H-*Tom*-*Gfp* and C3H-*Tom*-*Egr1* cells. * indicate the *p* values of gene expression levels in C3H-*Tom*-*Gfp* or C3H-*Tom*-*Egr1* at the first day of gene detection versus differentiation, with ****P < 0.0001; # indicate the *p* values of gene expression levels in C3H-*Tom*-*Gfp* versus C3H-*Tom*-*Egr1* for each time point, with ^##^*P* < 0.01, ^###^*P* < 0.001, ^####^*P* < 0.0001.

To further characterize the role of *Egr1* during adipocyte differentiation and confirm the results obtained with mASC, we used a second in vitro approach, taking advantage of the Tol2 genomic system to perform *Egr1* gain-of-function experiments in C3H10T1/2 mesenchymal cells. The Tol2 system was used in combination with the viral T2A system, which allowed stable and bi-cistronic expression of two proteins^30^ (Supplementary Fig. 4). Control C3H-*Tom*-*Gfp* cells co-expressed cytoplasmic Tomato and nuclear H2B-GFP fluorescent reporter proteins, whereas in C3H-*Tom*-*Egr1* cells, the *H2B-Gfp* coding sequence was replaced with *Egr1* coding sequence (Supplementary Fig. 4). C3H-*Tom*-*Gfp* and C3H-*Tom*-*Egr1* cells exhibited cytoplasmic *Tomato* expression and nuclear *H2B-Gfp* or *Egr1* expression, respectively (Figs. 5A and 6A).

At the end of white adipogenic stimulation, 90% of C3H-*Tom*-*Gfp* and 95% of C3H-*Tom*-*Egr1* cells differentiated into white adipocytes, marked by the appearance of large lipid droplets in the cytoplasm (Fig. 5B, C, white arrowheads). These observations indicate that *Egr1* gain-of-function promotes C3H10T1/2 cell differentiation into white adipocytes and support previous findings showing increased *Egr1* expression in WAT of obese mice and humans compared to lean individuals^22,23^. In contrast, while 97% of C3H-*Tom*-*Gfp* differentiated into beige adipocytes, indicated by the presence of small lipid droplets (Fig. 6B, C, white arrowheads), only 50% of C3H-*Tom*-*Egr1* cells achieved beige differentiation. These results confirm that *Egr1* gain-of-function in C3H10T1/2 mesenchymal stem cells reduces beige adipocyte differentiation^20^.

To further characterize the consequences of *Egr1* gain of function during white and beige differentiation, we quantified the expression of *Egr1* and several generic (*Cebpb*, *Pparg*, *Retn*, *Dcun1d3*) and beige (*Ucp1*, *Cidea*, *Cox8b*) adipocyte markers in C3H-*Tom*-*Gfp* and C3H-*Tom*-*Egr1* cells. As expected, C3H-*Tom*-*Egr1* cells display stable overexpression of *Egr1* during white (Fig. 5D) and beige (Fig. 6D) adipocyte differentiation, compared to control C3H-*Tom*-*Gfp* cells. The generic adipogenic factors *Cebpb* and *Pparg* were up regulated during white (Fig. 5D) and beige (Fig. 6D) adipocyte differentiation in both control C3H-Tom *Gfp* and C3H-Tom-*Egr1* cells. *Egr1* gain-of-function did not affect *Cebpb* and *Pparg* expression. These results indicate that while Egr1 is required to repress *Cebpb*^20^ (Fig. 4D) and *Pparg* expression (Fig. 4D) during mASC differentiation, it is not sufficient to affect their expression during adipocyte differentiation of C3H10T1/2 cells (Figs. 5D and 6D).

*Ucp1* was not detected at the end of white differentiation (Fig. 5D). As expected, the expression of the beige markers *Ucp1, Cidea* and *Cox8b* was upregulated in control C3H-*Tom*-*Gfp* cells upon differentiation into beige adipocytes (Fig. 6D). A moderate decrease in *Ucp1* expression was also observed in C3H-*Tom*-*Egr1* cells when compared to control cells (Fig. 6D), in accordance with their impaired beige differentiation. Surprinsingly, an increase of both *Cidea* and *Cox8b* expression was also observed during white adipocyte differentiation (Fig. 5D). In addition, *Egr1* gain of-function increases both *Cidea* and *Cox8b* expression during white (Fig. 5D) or beige (Fig. 6D) adipocyte differentiation. Egr1 therefore acts as an activator of *Cidea* and *Cox8b* expression during white and beige adipocyte differentiation of C3H10T1/2 and is able to repress *Ucp1* expression only. One hypothesis could be that, in contrast to the in vivo context (Fig. 2) the co-factors allowing Egr1 to repress *Cox8b* and *Cidea* might not be expressed by C3H10T1/2 cells.

Both *Retn* and *Dcun1d3* expression were upregulated in C3H-*Tom*-*Gfp* cells after white and beige differentiation (Figs. 5D and 6D), which strengthens the hypothesis of a generic role of these two genes during adipocyte differentiation. In addition, *Dcun1d3* expression was not altered in C3H-*Tom*-*Egr1* cells compared to controls (Figs. 5D and 6D), showing that *Egr1* is not required to regulate *Dcun1d3* expression during adipocyte differentiation. We could not detect any expression of the white adipocyte marker *Lep* either in C3H-*Tom*-*Gfp* or in C3H-*Tom*-*Egr1* cells during white adipocyte differentiation. These results highlights the limitations of in vitro cell lines in the analysis of white adipocyte differentiation and confirms that primary mASC are the only in vitro system that allows *Lep* detection^31^.

Collectively, these results indicate that *Egr1* is sufficient to promote white adipocyte differentiation of C3H10T1/2 cells and to impair beige differentiation, presumably by down-regulation of *Ucp1* expression.

**Figure 7:**
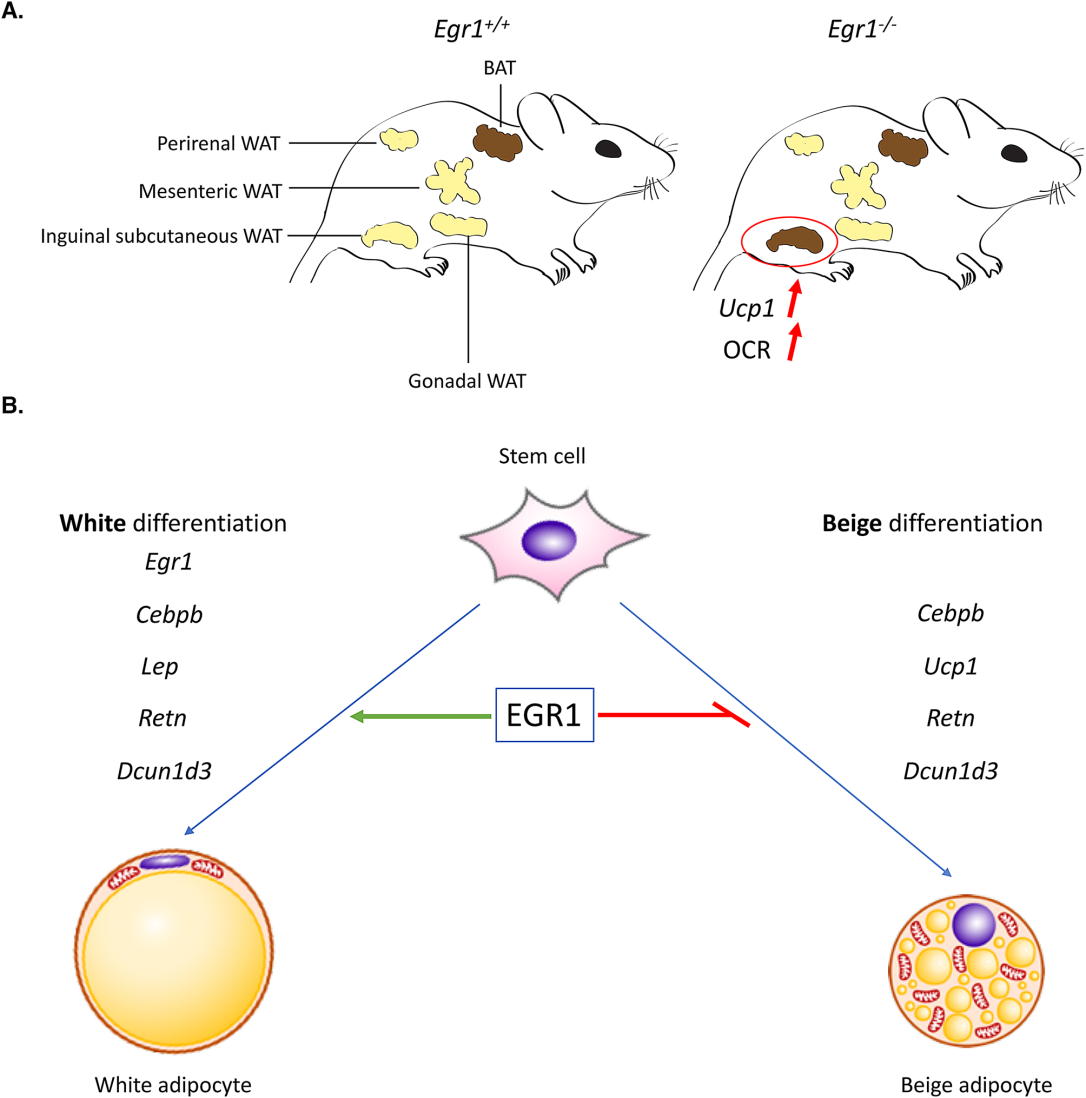
Schematic representation of the consequences of *Egr1* loss or gain-of-function on adipose tissue metabolism and adipocyte differentiation. (**A**) *Egr1* depletion leads to spontaneous and specific browning of inguinal subcutaneous white adipose tissue (WAT), visualized by increased *Ucp1* expression and oxygen consumption rate (OCR). (**B**) *Egr1* is necessary and sufficient to promote white adipocyte differentiation and reduce beige differentiation through the regulation of *Egr1*, *Cebpb*, *Ucp1*, *Lep*, *Retn* and *Dcun1d3* expression.

In summary, *Egr1* deletion induces spontaneous WAT browning specifically in the inguinal subcutaneous depot. This process acts through *Ucp1* upregulation and results in OCR increase at a level close to the BAT (Fig. 7). In vitro differentiation of mASC isolated from *Egr1* mutant SC-WAT confirmed that *Egr1* loss-of-function promotes beige adipocyte differentiation through *Ucp1* upregulation, and repress white adipocyte differentiation. We confirmed these results using *Egr1* gain-of-function experiments in C3H10T1/2 mesenchymal stem cells. Altogether, our study reveals that *Egr1* is necessary and sufficient to promote full white adipocyte differentiation and sufficient to prevent beige adipocyte differentiation. These results are in accordance with the increased expression of *Egr1* observed in obese mice and humans^22,23^ and establishes *Egr1* as a putative promising target to counteract obesity.

## Methods

### Mouse line

All experimental procedures using mice were conducted in accordance with the European guidelines (2010/63/UE) and were approved by the French National Ethic Committee for animal experimentation No. 05 and are registered under the number 01789.02.

*Egr1* gene was inactivated in C57BL/6 J mice by insertion of the *LacZ* coding sequence within the *Egr1* 5′ untranslated region, in addition to a frameshift mutation upstream of the DNA-binding domain of *Egr1*^32^. The line was maintained on a C57BL/6J genetic background (JANVIER, France). Animals were bred under controlled photo-period (lights on 08:00–20:00 h), chow diet and tap water ad libitum.

### RNA extraction from fat pads

Fresh inguinal subcutaneous (WAT), brown (BAT), gonadic (GAT), mesenteric (MAT) and perirenal (PAT) fat pads were removed from 8-month-old euthanized *Egr1*^+*/*+^ and *Egr1*^*-/-*^ female mice and homogenized using a mechanical disruption device (Lysing Matrix A, Fast Prep MP1, 4 × 30 s, 6 m s^−1^). Total RNA was isolated using the RNeasy mini kit (QIAGEN), with 15 min of DNase I (QIAGEN) treatment as previously described^20^.

### In situ hybridization to adipose tissue sections

The mouse *Dcun1d3* fragment was amplified by PCR (reverse 5′ggtcatcactagccatcctaac; forward 5′cagtattcagggagggactttg) from SC-WAT cDNA and cloned in the PGEM-T easy plasmid (PROMEGA). The antisense probe was synthesized using T7 polymerase after SalI digestion. The sense probe was obtained using Sp6 polymerase after SacII digestion. Inguinal subcutaneous fat pads were isolated from 1-month-old female mice, fixed in 4% paraformaldehyde overnight and cryo-sectioned. 8 µm wax tissue sections were used for in situ hybridization as previously described^33^.

### Quantification of mitochondrial DNA

WAT and BAT were dissected from 8-months-old control *Egr1*^+*/*+^ and mutant *Egr1*^*-/-*^ female mice and immediately frozen at −80 °C. DNA purification was performed using the QIAmp DNA Minikit (QIAGEN). DNA concentration and purity were measured using a Nanodrop. Quantitative real time PCR was performed on a StepOnePlus PCR machine (APPLIED BIOSYSTEMS) using the FastStart Universal SYBR Green Master (ROCHE) with primers that amplify the mitochondrial *CytB* gene and the nuclear encoded *NDUFV1* gene as endogenous reference, as described in^34^.

### Metabolic and bio-energetic analysis

Tissue bioenergetics were analyzed on a Seahorse extracellular flux analyzer (XF^e^24) according to the manufacturer's instructions. 2 mg explants of WAT and BAT freshly dissected from 8-months-old control *Egr1*^+*/*+^ and mutant *Egr1*^*−/−*^ female mice were immediately placed in a Seahorse XF^e^24 islet plate in 500 µL of pre-heated minimal assay medium (SEAHORSE BIOSCIENCES, 37 °C, pH 7.40) supplemented with glucose (2.25 g/L), sodium pyruvate (1 mM) and L-glutamine (2 mM). The tissue weight was based on initial assays that optimized the oxygen consumption rate (OCR). After one hour of equilibration at 37 °C in a non-CO_2_ incubator, OCR was measured successively in control condition (basal rates) and after successive injections of oligomycin (1 μM) that inhibits ATP synthase (ATP-linked OCR), FCCP (10 μM) that uncouples mitochondrial OXPHOS (maximal OCR) and mixed rotenone/antimycin (10 μM) that inhibit complexes I and III (non-mitochondrial OCR).

### Detection and quantification of carbonylated proteins

WAT and BAT were dissected from 8-months-old control *Egr1*^+*/*+^ and mutant *Egr1*^*-/-*^ female mice and immediately frozen at -80 °C. The day of extraction, tissues were homogenized in lysis buffer (8 M urea, 2 M thiourea, 4% CHAPS and 10 mM DTT). After 30 min on ice, lysates were clarified by centrifugation at 20,000* g* for 15 min at 4 °C. Protein concentrations of the supernatants were determined by the Bradford method. Labeling of protein carbonyl groups was obtained by overnight incubation of 20 µg protein extracts with CF555DI hydrazide (BIOTIUM, Fremont, CA, USA) at 4 °C under medium agitation. Labeled samples were then resolved by SDS-PAGE in an anykD pre-cast gel (BIORAD). After three washes using a solution 7% acetic acid and 10% ethanol, CF555DI hydrazide labeling was detected using a ChemiDoc MP Imagin System (BIORAD). Total protein staining was performed using InstantBlue Protein Stain (EXPEDEON). Raw data were analyzed using the Fiji/ImageJ software. In both CF555DI and Coomassie’s analysis, the protein profiles were quantified by measuring the intensity of the lane for each sample. Data were normalized using the CF555DI/Coomassie ratio.

### Isolation of murine adipose stem/stromal cells (mASC)

mASC were isolated as previously described^35^. Briefly, subcutaneous inguinal fat pads were excised from wild type or *Egr1*^*−/−*^ female mice, minced in prewarmed Krebs–ringer bicarbonate KRB (118 mM NaCl, 5 mM KCL, 2.5 mM CaCl_2_, 2 mM KH_2_PO_4,_ 2 mM MgSO_4_, 25 mM NaHCO_3_, 5 mM glucose, pH = 7.4) and digested in collagenase solution (0.1%). After centrifugation, the stromal vascular fraction (SVF) was rinsed in PBS, resuspended in prewarmed Stromal medium (Dulbecco’s Modified Eagle’s Medium (DMEM; GIBCO) supplemented with 10% FBS, 1% penicillin–streptomycin) and seeded on plastic flasks to allow mASC adhesion. Cells were cultured at 37 °C in humidified atmosphere with 5% CO2.

### Plasmid cloning

The *Egr1* coding sequence was obtained by PCR amplification of the plasmid *pCAß-Egr1*^36^ using the following primers: m*Egr1*-Bstbl-Forward-CTAATGTTCGAAATGGCAGCGGCCAAGGC, m*Egr1*-Pmll-Reverse-ATCGTCACGTGTATTAGCAAATTTCAAT, and cloned in the transposable *pT2AL-CMV-Tom-T2A-Gfp* plasmid^30^. The *H2B*-*Gfp* coding sequence was removed from *pT2AL-CMV-Tom-T2A-Gfp* by BstbI and PmlI digestion and replaced with *Egr1* coding sequence.

### Transfection of C3H10T1/2 cells and isolation of C3H-*Tom*-*Gfp and* C3H-*Tom*-*Egr1* cells

Mouse mesenchymal stem cells C3H10T1/2^37^ were seeded to 6-well plates at a density 20.000 cells/cm2 in stromal medium (Dulbecco’s Modified Eagle’s Medium (DMEM; GIBCO) supplemented with 10% FBS, 1% penicillin–streptomycin). Cells were transfected at 80% confluence by lipofectamine P3000 (Invitrogen) with 1 µg of plasmid encoding the transposase (pT2TP) and 2 µg of *pT2AL-CMV-Tom-T2A-Gfp*, or *pT2AL-CMV-Tom-T2A-*Egr1, in order to obtain stable C3H-*Tom*-*Gfp* and C3H-*Tom*-*Egr1* cells that overexpress *GFP* and *Egr1*, respectively. Two days after transfection, C3H-*Tom*-*Gfp* and C3H-*Tom*-*Egr1* cells were resuspended into Fluorescence Analysis Cell sorting (FACS) suspension medium (Sterile PBS 1X, 10% FBS, 1% P/S, 2 mM EDTA and DAPI) and purified on a FACS Aria 3 (BECTON DICKINSON, CA). Live cells were gated based on the morphology, doublets were excluded and cells were sorted based on their Tomato expression.

### Cell cultures

Mouse adipose stem/stromal cells mASC-*Egr1*^+*/*+^ and mASC-*Egr1*^*-/-*^, or mouse mesenchymal stem cells C3H-*Tom*-*Gfp* and C3H-*Tom*-*Egr1* were plated on 6-well plates at a density of 7.500 cells/cm^2^ in Dulbecco’s Modified Eagle’s Medium (DMEM, INVITROGEN) supplemented with 10% foetal bovine serum (FBS, Sigma), 1% penicillin–streptomycin (SIGMA), 1% Glutamine (SIGMA), 800 μg/ml G418 Geneticin (SIGMA) and incubated at 37 °C in humidified atmosphere with 5% CO_2_ as described in^20^. Cells were cultured in white adipocyte differentiation medium (STEMPRO, THERMOFISHER) until differentiation which takes 7 to 12 days. The medium was changed every 3–4 days. Day 0 corresponds to the addition of white differentiation medium, at 100% confluence. mASC-*Egr1*^+*/*+^ and mASC-*Egr1*^*-/-*^ subjected to white adipocyte differentiation medium were stopped at Day 0, Day 4, Day 8 and Day 10 or 12 for gene expression analysis and stopped at Day 10 for Oil Red O staining (as described in previous study^20^. C3H-*Tom*-*Gfp* and C3H-*Tom*-*Egr1* cells subjected to white adipocyte differentiation medium were stopped at Day 0 and at the end of differentiation for gene expression analysis.

Confluent cells were cultured in beige differentiation induction medium for 2 days and in beige maturation medium for 7 to 10 days according to published protocols^20,38^. Day 0 corresponds to the addition of beige differentiation induction medium at confluence. The maturation medium was changed every 3–4 days. mASC-*Egr1*^+*/*+^ and mASC-*Egr1*^*-/-*^ subjected to beige adipocyte differentiation medium were fixed for Oil Red O staining at Day 10 or lysed for gene expression analysis at Day 0, Day 4, Day 8 and Day 10.

C3H-*Tom*-*Gfp* and C3H-*Tom*-*Egr1* cells subjected to beige adipocyte differentiation medium were stopped at Day 0 and at the end of differentiation for gene expression analysis.

Cell number measurements were performed using the free software Fiji^39^.

### Reverse-transcription and quantitative real time PCR

Total RNAs extracted from mouse fat pads, mASC-*Egr1*^+*/*+^ and mASC-*Egr1*^*-/-*^, or C3H-*Tom*-*Gfp* and C3H-*Tom*-*Egr1* cells were Reverse Transcribed using the High Capacity Retro-transcription kit (Applied Biosystems). Quantitative PCR analyses were performed using SYBR Green PCR Master Mix (Applied Biosystems) and primers listed in Supplementary Table 1. The relative mRNA levels were calculated using the 2^−∆∆Ct^ method^40^. The Cts were obtained from Ct normalized to *Rn18S* levels in each sample. For mRNA level analysis in fat pads, 7 to 9 independent RNA samples of 8-month-old *Egr1*^+*/*+^ and *Egr1*^*-/-*^ female mice were analysed in duplicate. For mRNA level analysis of mASC-*Egr1*^+*/*+^ and mASC-*Egr1*^*-/-*^ cultures, 5 to 12 independent RNA samples were analysed in duplicate for each time point. For mRNA level analysis of C3H10T1/2-*Tom*-*Gfp* and C3H10T1/2-*Tom*-*Egr1* cell cultures, 12 independent RNA samples were analysed in duplicate for each time point.

### Statistical analyses

Data was analysed using the non-parametric Mann-Withney test with Graphpad PRISM V6 or Student t test. Results are shown as means ± standard deviations. The *p* values are indicated either with the value or with * or #, as in^20^.

## Supplementary information

Supplementary information.
