# Supplementary material for: Egr1 loss-of-function promotes beige adipocyte differentiation and activation specifically in inguinal subcutaneous white adipose tissue
Source: Sci Rep. 2020 Sep 28;10:15842. doi: 10.1038/s41598-020-72698-w (PMC7522992; doi:10.1038/s41598-020-72698-w)
Supplement: Supplementary file 1 — Supplementary information. [file 41598_2020_72698_MOESM1_ESM.pdf]

Supplementary information

Egr1 loss-of-function promotes beige adipocyte differentiation and activation specifically in inguinal subcutaneous white adipose tissue

Marianne Bléher[1,$], Berbang Meshko[1,$], Isabelle Cacciapuoti[3,4], Rachel Gergondey[1,3], Yoann Kovacs[1,3], Delphine Duprez[1,2], Aurore L'Honoré[1,3,#], Emmanuelle Havis[1,2,#,*].

[1] Sorbonne Université, F75005 Paris, France

[2] French National Centre for Scientific Research (CNRS) UMR7622, Institute of Biology Paris Seine-Developmental biology laboratory, Inserm U1156, F75005 Paris, France

[3] French National Centre for Scientific Research (CNRS) UMR8256, Institute of Biology Paris Seine -Integrative cellular ageing and inflammation, F-75005 Paris, France.

[4] Inovarion, Paris, France

[$] Co-first authors

[#] Co-senior authors

*Corresponding author: Emmanuelle Havis, emmanuelle.havis@sorbonne-universite.fr, +33 1 44 27 34 52

Figure legends

Supplementary figure 1. *Egr1* loss-of-function has no effect on adipogenesis-related gene expression in Brown adipose tissue. BAT of 8-month-old *Egr1*$^{+/+}$ and *Egr1*$^{-/-}$ mice were used for RNA purification. RNA samples were analysed by RT-qPCR for *Egr1*, *Cebpb*, *Pparg*, *Ucp1*, *Lep* and *Dcun1d3* expression. Experiments were performed with n=8 animals for each genotype. Transcripts are shown relative to the level of *18S* transcripts, with control *Egr1*$^{+/+}$ being normalized to 1. The relative mRNA levels were calculated using the $2^{\wedge-\Delta\Delta Ct}$ method. The p-values were obtained using the Mann-Withney test, with * $p<0.05$, ** $p<0.01$, *** $p<0.001$.

Supplementary figure 2. *Egr1* loss-of-function has no effect on adipogenesis-related gene expression in Gonadic, Mesenteric and Perirenal white adipose tissues. (A) GAT, **(B)** MAT and **(C)** PAT of 8-month-old *Egr1*$^{+/+}$ and *Egr1*$^{-/-}$ mice were used for RNA purification. RNA samples were analysed by RT-qPCR for *Egr1*, *Cebpb*, *Pparg*, *Ucp1*, *Lep* and *Dcun1d3* expression. Experiments were performed with n=8 animals for each genotype. Transcripts are shown relative to the level of *18S* transcripts, with control *Egr1*$^{+/+}$ being normalized to 1. The relative mRNA levels were calculated using the $2^{\wedge-\Delta\Delta Ct}$ method. The p-values were obtained using the Mann-Withney test, with * $p<0.05$, ** $p<0.01$, *** $p<0.001$.

Supplementary figure 3. *Egr1* loss-of-function does not affect GAT metabolic activity. (A) Gonadal adipose tissues (GAT) of 8-month-old control *Egr1*$^{+/+}$ and mutant *Egr1*$^{-/-}$ female mice were dissected and used for DNA purification. Mitochondrial (*Cyt B*) and nuclear (*Ndufv1*) genes were quantified by qPCR and histogram represents their ratio. Error bars represent the means + standard deviations with n=3 animals for each genotype, *$p<0.05$. **(B, C)** Mitochondrial respiration, measured by oxygen consumption rate (OCR) in basal conditions, and after sequential addition of Oligomycin, FCCP, and a mix of Rotenone/Antimycin were simultaneously recorded on GAT tissues, freshly dissected from 8-month-old control *Egr1*$^{+/+}$ and mutant *Egr1*$^{-/-}$ female mice. **(C)** Histogram represents the basal OCR (determined as the difference between OCR before oligomycin and OCR after rotenone/antimycin A), maximal OCR (difference between OCR after FCCP and OCR after rotenone/antimycin A), ATP-linked OCR (difference between OCR before and after oligomycin), and the non-mitochondrial OCR (OCR after rotenone and antimycin A

treatment) calculated from data obtained in B. (E) WAT and BAT dissected from 8-month-old control *Egr1*$^{+/+}$ and mutant *Egr1*$^{-/-}$ female mice were used to determine carbonylated protein levels.

Supplementary figure 4. Strategy used for *Egr1* gain-of-function in C3H10T1/2 cells. (A) Schematic representation of the T2A control vector characterized by the presence of the two reporter fluorescent genes *Tomato* and *H2B-GFP*, separated by the T2A peptide and flanked by the Tol2 genomic integration system. In this vector, the *H2B-GFP* coding sequence has been replaced by the *Egr1* coding sequence to allow *Egr1* overexpression. **(B)** Strategy used for stable and bi-cistronic expression of *Tomato* and *H2B-GFP* or *Egr1*. C3H10T1/2 cells were co-transfected with a first vector containing the transposase coding sequence and with the T2A-H2B-GFP or the T2A-Egr1 vector. Transposase expression leads to stable integration of the CMV/βactin promoter-Tomato-T2A-H2B-GFP or CMV/βactin promoter-Tomato-T2A-EGR1 transgenes into the cell genome. Expression of both Tomato-T2A-H2B-GFP and Tomato-T2A-EGR1 cassettes are under the control of the CMV/βactin promoter and leads to the transcription of one single mRNA. During translation, the self-cleavage of the T2A peptide allows the production of the two proteins Tomato and H2B-GFP or Tomato and EGR1 in stoichiometric proportions. Tomato and H2B-GFP localizations are cytoplasmic and nuclear, respectively [31].

Supplementary Table 1. List of primers used for RT-qPCR analysis

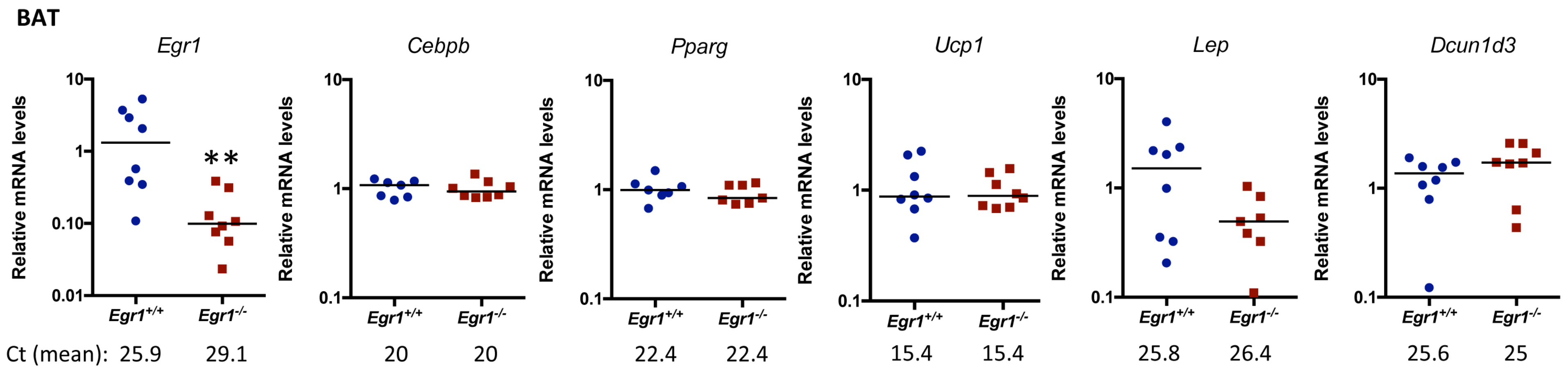
BAT
Egr1
Cebpb
Pparg
Ucp1
Lep
Dcun1d3
Relative mRNA levels
10
1
0.10
0.01
0.1
**
Egr1+/+
Egr1-/-
Ct (mean): 25.9 29.1
20 20
22.4 22.4
15.4 15.4
25.8 26.4
25.6 25

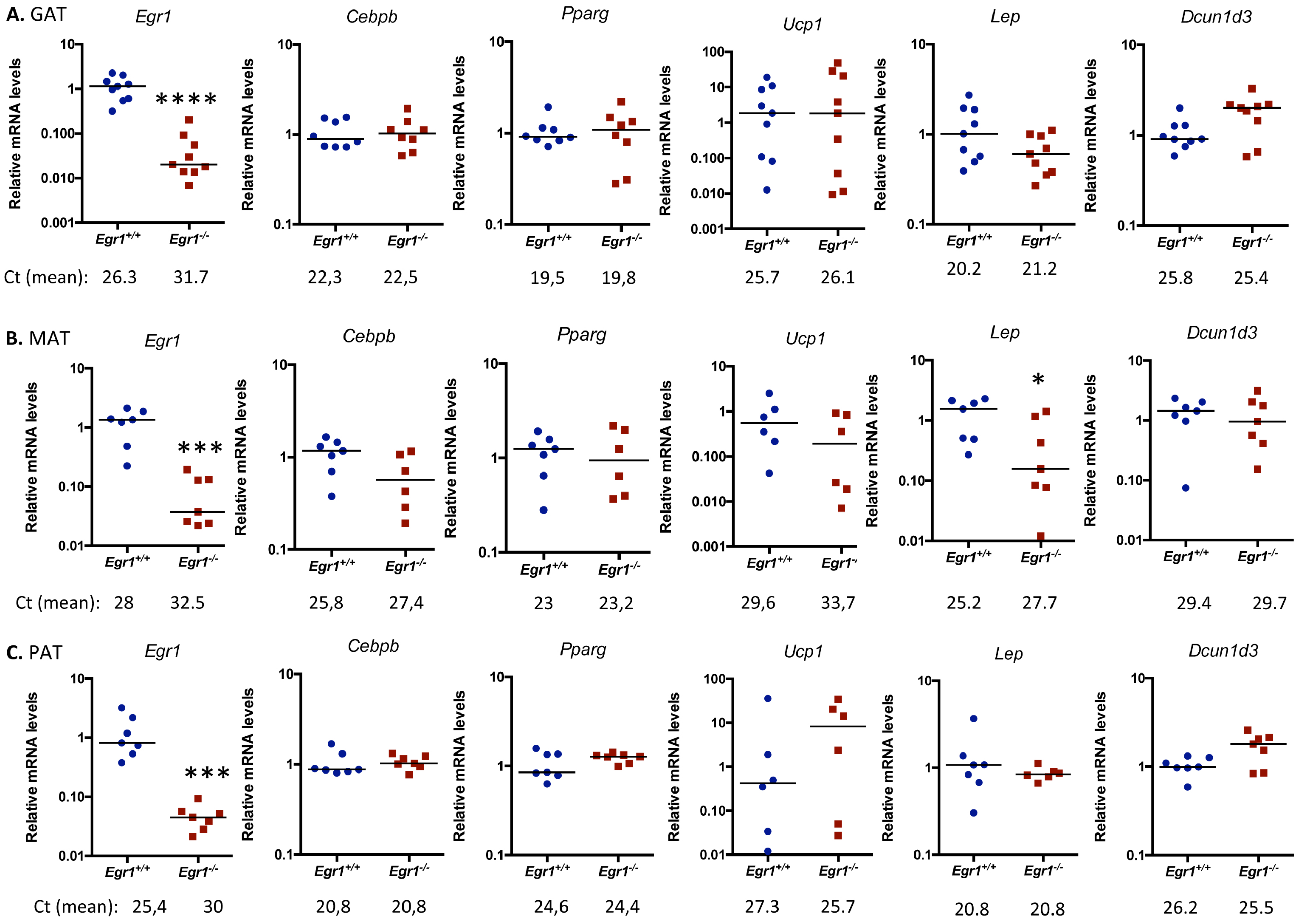

A. GAT
Egr1
Cebpb
Pparg
Ucp1
Lep
Dcun1d3
Relative mRNA levels
****
Egr1+/+
Egr1-/-
Ct (mean): 26.3 31.7
22,3 22,5
19,5 19,8
25.7 26.1
20.2 21.2
25.8 25.4
B. MAT
Egr1
Cebpb
Pparg
Ucp1
Lep
Dcun1d3
***
*
Egr1-
Ct (mean): 28 32.5
25,8 27,4
23 23,2
29,6 33,7
25.2 27.7
29.4 29.7
C. PAT
Egr1
Cebpb
Pparg
Ucp1
Lep
Dcun1d3
***
Ct (mean): 25,4 30
20,8 20,8
24,6 24,4
27.3 25.7
20.8 20.8
26.2 25.5

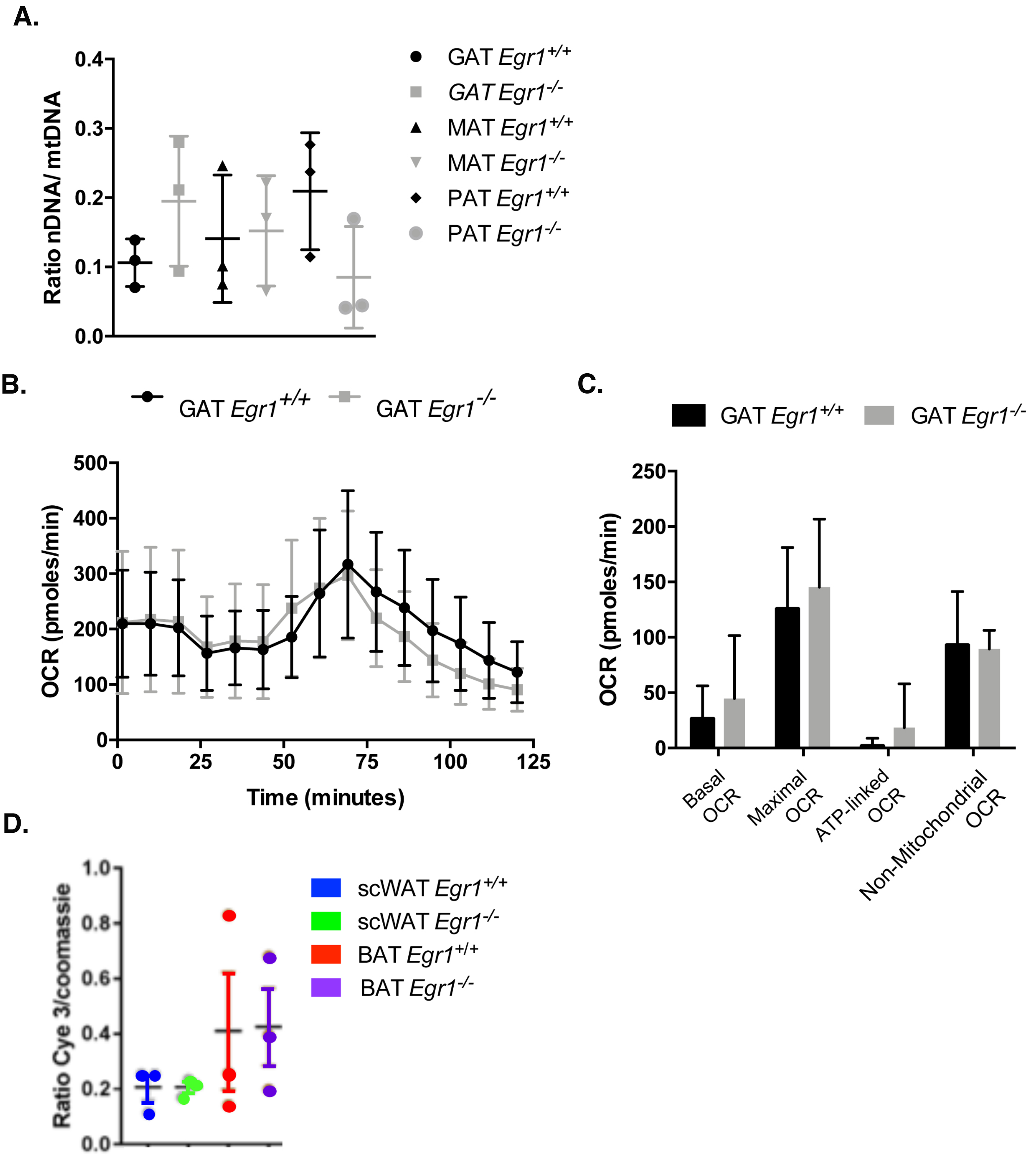

A.
Ratio nDNA/ mtDNA
0.0
0.1
0.2
0.3
0.4
GAT Egr1+/+
GAT Egr1-/-
MAT Egr1+/+
MAT Egr1-/-
PAT Egr1+/+
PAT Egr1-/-
B.
GAT Egr1+/+
GAT Egr1-/-
OCR (pmoles/min)
0
100
200
300
400
500
0
25
50
75
100
125
Time (minutes)
C.
GAT Egr1+/+
GAT Egr1-/-
OCR (pmoles/min)
0
50
100
150
200
250
Basal OCR
Maximal OCR
ATP-linked OCR
Non-Mitochondrial OCR
D.
Ratio Cye 3/coomassie
0.0
0.2
0.4
0.6
0.8
1.0
scWAT Egr1+/+
scWAT Egr1-/-
BAT Egr1+/+
BAT Egr1-/-

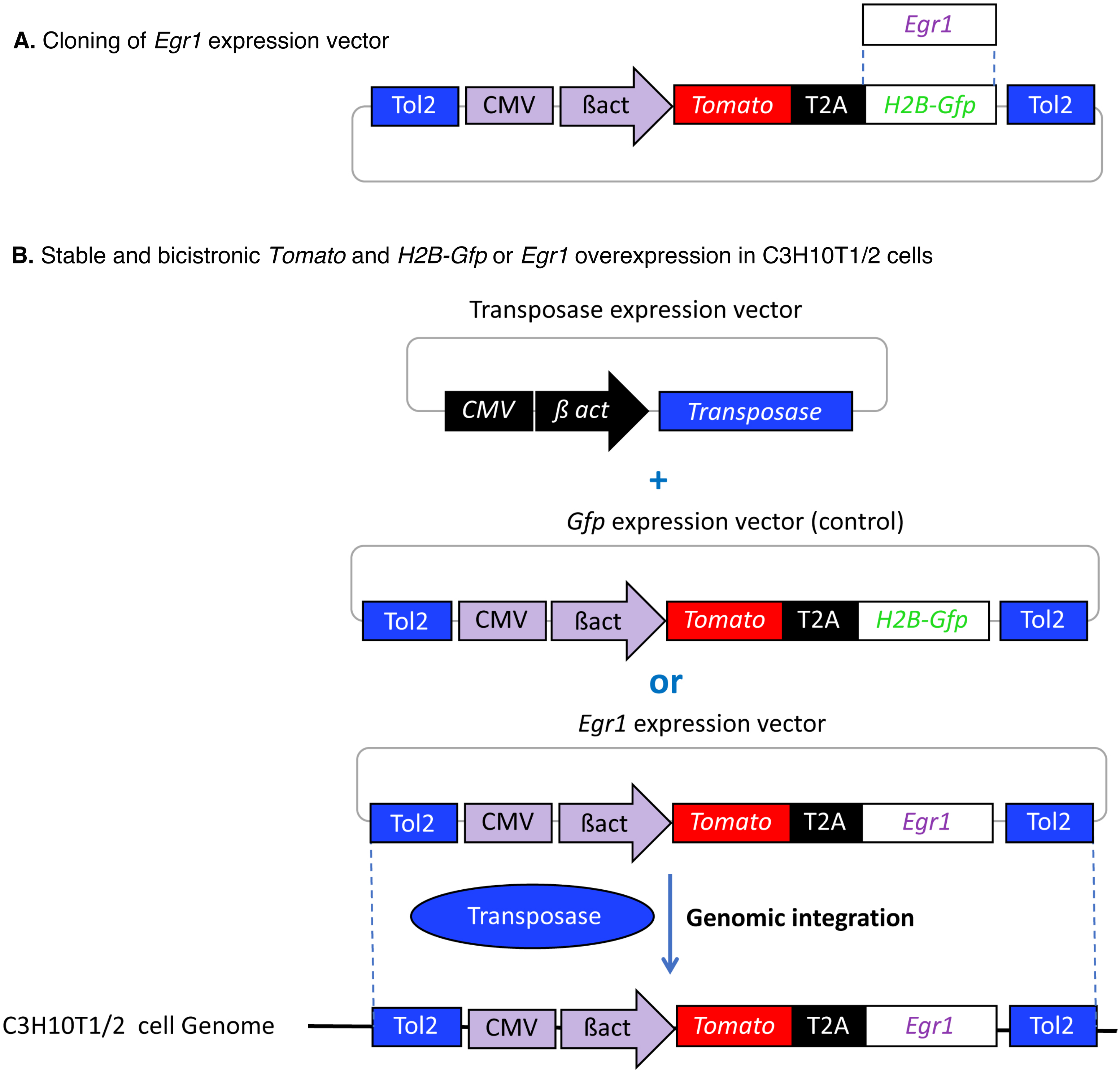
A. Cloning of Egr1 expression vector
Egr1
Tol2
CMV
ßact
Tomato
T2A
H2B-Gfp
Tol2
B. Stable and bicistronic Tomato and H2B-Gfp or Egr1 overexpression in C3H10T1/2 cells
Transposase expression vector
CMV
ß act
Transposase
+
Gfp expression vector (control)
Tol2
CMV
ßact
Tomato
T2A
H2B-Gfp
Tol2
or
Egr1 expression vector
Tol2
CMV
ßact
Tomato
T2A
Egr1
Tol2
Transposase
Genomic integration
C3H10T1/2 cell Genome
Tol2
CMV
ßact
Tomato
T2A
Egr1
Tol2

**Primers for RT-qPCR analysis**		
**Gene name**	**Forward Primer**	**Reverse Primer**
Cebpb	5’-ACGACTTCCTCTCCGACCTCT	5’-CGAGGCTCACGTAACCGTAGT
Cidea	5’- ACTTCCTCGGCTGTCTCAATGTCA	5’- TCAGCAGATTCCTTAACACGGCCT
Cox8b	5’- AGCCAAAACTCCCACTTCC	5’- TCTCAGGGATGTGCAACTTC
Dcun1d3	5’-GCTGACTCTGTCCTACCTTATTC	5’-CCACCCACTTTCCAGTACAT
Dio2	5’-CTTCTCTACCACCACCTTC	5’- CATCTTCACCCAGTTTAACC
Egr1	5’-CAGCGCCTTCAATCCTCAAG	5’-GCGATGTCAGAAAAGGACTCTGT
Lep	5’-TACCGCATTTCAGGGCACAT	5’- CCCAGGTATCCCGTGTCAAC
Pparg	5’-TCGGTGATGCACTGCCTATG	5’-GAGAGGTCCACAGAGCTGATT
Retn	5’-GCCATCGACAAGAAGATCAA	5’-CTTCCCTCTGGAGGAGACTG
Rn18S	5’-GGCGACGACCCATTCG	5’-ACCCGTGGTCACCATGGTA
Rplp0	5’- ACCTCCTTCTTCCAGGCTTT	5’- CTCCCACCTTGTCTCCAGTC
Ucp1	5’- GGGCATTCAGAGGCAAATCAGCTT	5’- ACACTGCCACACCTCCAGTCATTA
